# Impact of Combined Clenbuterol and Metoprolol Therapy on Reverse Remodelling during Mechanical Unloading

**DOI:** 10.1371/journal.pone.0092909

**Published:** 2014-09-30

**Authors:** Manoraj Navaratnarajah, Urszula Siedlecka, Michael Ibrahim, Carin van Doorn, Gopal Soppa, Ajay Gandhi, Adarsh Shah, Punam Kukadia, Magdi H. Yacoub, Cesare M. Terracciano

**Affiliations:** Harefield Heart Science Centre, Imperial College London, National Heart and Lung Institute, Laboratory of Cellular Electrophysiology, Harefield Hospital, Harefield, Middlesex, United Kingdom; University of Louisville, United States of America

## Abstract

**Background:**

Clenbuterol (Cl), a β_2_ agonist, is associated with enhanced myocardial recovery during left ventricular assist device (LVAD) support, and exerts beneficial remodelling effects during mechanical unloading (MU) in rodent heart failure (HF). However, the specific effects of combined Cl+β_1_ blockade during MU are unknown.

**Methods and Results:**

We studied the chronic effects (4 weeks) of β_2_-adrenoceptor (AR) stimulation via Cl (2 mg/kg/day) alone, and in combination with β_1_-AR blockade using metoprolol ((Met), 250 mg/kg/day), on whole heart/cell structure, function and excitation-contraction (EC) coupling in failing (induced by left coronary artery (LCA) ligation), and unloaded (induced by heterotopic abdominal heart transplantation (HATx)) failing rat hearts. Combined Cl+Met therapy displayed favourable effects in HF: Met enhanced Cl's improvement in ejection fraction (EF) whilst preventing Cl-induced hypertrophy and tachycardia. During MU combined therapy was less beneficial than either mono-therapy. Met, not Cl, prevented MU-induced myocardial atrophy, with increased atrophy occurring during combined therapy. MU-induced recovery of Ca^2+^ transient amplitude, speed of Ca^2+^ release and sarcoplasmic reticulum Ca^2+^ content was enhanced equally by Cl or Met mono-therapy, but these benefits, together with Cl's enhancement of sarcomeric contraction speed, and MU-induced recovery of Ca^2+^ spark frequency, disappeared during combined therapy.

**Conclusions:**

Combined Cl+Met therapy shows superior functional effects to mono-therapy in rodent HF, but appears inferior to either mono-therapy in enhancing MU-induced recovery of EC coupling. These results suggest that combined β_2_-AR simulation +β_1_-AR blockade therapy is likely to be a safe and beneficial therapeutic HF strategy, but is not as effective as mono-therapy in enhancing myocardial recovery during LVAD support.

## Introduction

The use of LVADs in end stage HF continues to expand [1]. LVADs are used primarily as “bridge to transplantation”. In a minority of patients they have been used as a “bridge to recovery” (BTR), where sufficient functional recovery of the failing heart is achieved, allowing device removal. However, explantation rates remain universally low (≤10%) [2], [3].

HF-associated remodelling is a complex series of whole heart, cellular, molecular, structural and functional changes affecting various pathways, including EC coupling, the extracellular matrix (ECM), apoptosis and adrenergic receptor signalling [4]. Previously thought to be uni-directional, such adverse remodelling is now considered reversible, a process termed reverse remodelling. Pharmacological intervention induces mild reversal of HF remodelling, demonstrating functional and prognostic benefit [5]–[7], however, a more substantial degree of reverse remodelling is achieved via LVAD [8]. Despite profound LVAD-induced reverse remodelling, functional myocardial recovery is rare. This disparity is proposed to arise due to negative MU-induced changes occurring during prolonged unloading [9], [10]. Indeed, in animals and humans, initial contractile recovery of failing hearts is seen to subsequently worsen during prolonged MU [3], [11]. Time dependent worsening of EC coupling [12], Ca^2+^ handling [11] and myocardial atrophy [13] are proposed negative drivers of contractile performance.

“Combination therapy” describes a strategy in which pharmacotherapy is combined with LVAD support, to enhance LVAD–induced positive remodelling and/or combat LVAD-induced “negative” remodelling [10]. In our institute this strategy - the “Harefield Protocol” - successfully enhanced rates of functional recovery (>63%) in patients with dilated cardiomyopathy [14], [15]. In this unique protocol, the β_2_-adrenoceptor (AR) agonist Cl was employed alongside conventional HF pharmacotherapy, primarily as an agent to prevent myocardial atrophy. Cl's ability to induce physiological cardiac hypertrophy [16] mandated its inclusion in this protocol, but whether anti-atrophic properties contributed to enhancing functional recovery is unclear [17]. Hence, Cl's precise role in enhancing functional recovery remains poorly defined and controversial. Beyond hypertrophic effects, Cl demonstrates other wide-ranging favourable whole heart/cellular functional, structural, metabolic, cell-survival, EC coupling and gene expression effects in non-failing [16], [18], failing [19], [20] and unloaded [19], [21] hearts.

BTR remains controversial, but the clinical success of combination therapy (14, 15) justifies continued scrutiny of this strategy. Therefore, the search for other novel pharmacological strategies aimed at enhancing LVAD-induced functional recovery continues, and we have recently shown the pacemaker current inhibitor ivabradine and Met to show positive effects on myocardial size, EC coupling and the ECM during mechanical unloading in rodent HF [22].

The efficacy of β-AR blockade in HF therapy is established [4], [6], [23] and is predominantly secondary to blockade of detrimental β_1_-AR signalling [4]. Greater understanding of the roles played by β_1_-/β_2_-AR signalling indicates distinct and often opposing physiological and pathological actions on cardiac structure and function [24], with chronic β_1_-AR signalling associated with pro-apoptotic and maladaptive remodelling [25] and chronic β_2_-AR signalling with cardio-protective actions, improved function and myocyte viability [26], [27]. Supporting this, combined β_1_-AR blockade (Met) and β_2_-AR stimulation (fenoterol) has shown synergistic beneficial remodelling effects in rodent HF [24], although lack of synergy (Cl+Met) has also been demonstrated [20]. These studies instituted pharmacotherapy early after LCA ligation (<3 weeks) and combined drug effects in an established rodent HF model remain unknown. β_2_-AR stimulation (Cl) demonstrates positive effects during MU in rodent studies [19], [28], however, the specific effects of combined β_2_-AR stimulation and β_1_-AR blockade remain unstudied within this setting.

In this study, we report for the first time the chronic effects of combined β_2_-AR stimulation (Cl) and β_1_-AR blockade (Met) on reverse remodelling during MU in a rodent HF model. In particular, we have examined the drug effects on whole heart/cell size, function, EC coupling and T-tubule structure in a rat model of established HF, 12 weeks post LCA ligation, and during MU using HATx.

## Methods

### Ethics Statement

The investigation conformed to the Guide for the care and Use of Laboratory Animals published by the US National Institutes of Health (NIH publication No 85–23, revised 1996). Experiments were approved by the Harefield Heart Science Centre ethics review panel.

*(For extended methods please refer to Methods S1).*

### Animal models

#### Induction of heart failure

HF was induced via permanent LCA ligation as previously described [11], [19]–[22], [24]. Animals were anaesthetised with isoflurane (5% induction, 1.5% maintenance) mixed with O_2_ (2–3 L/min), intubated and mechanically ventilated via rodent ventilator, and a left thoracotomy performed. The pericardium was opened and heart visualised, the proximal LCA was identified and a suture (6–0 prolene) placed underneath the artery and lightly tied. Heamostasis was achieved and the chest closed. Sham operated animals underwent the identical protocol but the suture was not tied. Acute 24 hour mortality was 25% in the LCA ligation group and 0% in the sham group. Male syngeneic Lewis rats (10 weeks old ∼200 g, Harlan, UK) were used for all experiments to avoid the need for immunosuppression following subsequent heterotopic abdominal transplant (HATx) in the mechanical unloading (MU) groups. 1 week after surgery LV dimensions and function (ejection fraction (EF)) were assessed by transthoracic echocardiography (TTE) (Acuson Sequoia™ 256; Acuson, USA), as previously described [18]–[21], [24]. LAD ligation animals with an EF less than 40% were included in the study, as in our experience animals with baseline function greater than this were unlikely to develop severe dysfunction 12 weeks following surgery. 12 weeks following LCA ligation the presence of severe cardiac dysfunction was confirmed via repeat TTE, and animals were randomised to groups (n = 8). All groups had similar average EF and variability. 3 month mortality was 1% following LCA ligation and 0% in sham group. TTE was performed under general anaesthesia with isoflurane 1%, mixed with O_2_ (2–3 L/min) to provide adequate sedation but minimal cardiac depression. Experiments were not performed in non-failing hearts as these show an exaggerated atrophic response during unloading compared with failing hearts [29].

In order to test the effects of pharmacotherapy (Cl and/or Met) in combination with MU, failing hearts (12 weeks after LCA ligation) were transplanted into the abdomen of syngeneic male Lewis rats (weight ∼250 g) as previously described [11]–[13], [19], for a period of 4 weeks. In brief, both animals (recipient and donor) were anaesthetised with isoflurane (5% induction, 1% maintenance) mixed with O_2_ (2–3 L/min), and self-ventilated throughout the procedure. The failing heart was removed from the donor animal under cardioplegic arrest, and the donor aorta anastomosed to the recipient abdominal aorta and the donor pulmonary artery to the recipient inferior vena cava. Total ischaemic time was <40 minutes and operative mortality <4%, with no deaths occurring in any of the treatment groups during the 4 week treatment period.

Recipients were then assigned to 4 treatment groups: (1) Cl (Sigma, England) 2 mg/kg/day via subcutaneous infusion using osmotic minipump (Model 2002, Alzet), (2) Met (Sigma, England) 250 mg/kg/day in drinking water, (3) Met 250 mg/kg/day +Cl 2 mg/kg/day via drinking water and osmotic minipump respectively, (4) no drug treatment (MUHF+Cl, MUHF+Met, MUHF+MetCl and MUHF, respectively). Animals in Met-treated and MUHF group also received an osmotic minipump containing normal saline. All groups contained 8 animals and treatment duration was 4 weeks. At end of treatment Cl levels in blood were measured as previously described [18], with no difference between the different groups treated (in µM: HF+Cl = 0.17+/−0.01, n = 8; MUHF+Cl = 0.17+/−0.01, n = 8; HF+MetCl = 0.16+/−0.01; n = 8; MUHF+MetCl = 0.16+/−0.02, n = 8; p = 0.93).

In order to test the effects of Cl and Met in non-transplanted failing hearts, HF animals were randomly allocated to 4 treatment groups: (1) Cl 2 mg/kg/day, (2) Met 250 mg/kg/day, (3) combined Met+Cl treatment, (4) no drug treatment (HF+Cl, HF+Met, HF+MetCl and HF, respectively). Drug administration was via drinking water and osmotic minipump, exactly as described above. At the end of the treatment period *in vivo* heart function was measured via repeat TTE.

We have compared the effects of Met during mechanical unloading with those of ivabradine in a previously published study [22]. Therefore, we have included this data here for comparison with the effects of Cl and combined MetCl therapy. The data acquired from the Met group are contemporary and obtained in otherwise identical conditions to the data for the Cl and MetCl groups.

#### In-vivo assessment of heart rate

4 animals per treatment group received implantable ECG telemetry transmitters (CA-F40 Data Sciences International, Minneapolis, MN) on day 0 of the 4 week treatment period, for *in vivo* HR and arrhythmia studies. Animals underwent continuous 24 hour ECG recording at weekly intervals during the treatment period. *In vivo* ECG recordings were acquired using Dataquest ART 3.1 software (Data Sciences International, Minneapolis, MN), and offline HR/arrhythmia analysis performed using ECG-Auto 2.4 software (EMKA, France).

#### Animal anaesthesia, analgesia and euthanasia

Adequacy of anaesthesia during all procedures was monitored by loss of reflexes, degree of muscle relaxation and respiration rate. Respiration rate and body temperature were monitored throughout all procedures and body temperature maintained at 37°C, via heating mat. Analgesia was provided by subcutaneous (SC) injection of vetergesic (buprenorphine), just prior to skin incision. For LCA ligation and implantation of telemetry device a single dose of 0.03 mg/kg was utilised, and for heterotopic abdominal heart transplant both the donor and recipient received a single dose of 0.05 mg/kg. Post-operative condition of the rats was monitored, and repeat SC injection administered as required (0.01–0.05 mg/kg) every 12 hours during the first week post-operative week. At the end of the 4 week treatment period animals were sacrificed via schedule 1 cervical dislocation. In each group, 4 hearts were utilised for acute cellular experiments, and 4 for histological analysis.

### Cardiomyocyte studies

LV cardiomyocytes were isolated by standard enzymatic digestion for 8–10 minutes using collagenase (1 mg/ml, Worthington, USA) and hyaluronidase (0.6 mg/ml, Sigma, England) [19] and used within 6 h.

#### Assessment of sarcomere shortening and calcium handling

Simultaneous assessment of sarcomere shortening and Ca^2+^ handling in field stimulated cardiomyocytes was performed using an inverted microscope (TE 200), Ionoptix system (Ionoptix Corporation, USA) and the Ca^2+^ sensitive, single-excitation dual emission fluorescent dye Indo-1 AM (Molecular Probes, USA). In addition, sarcoplasmic reticulum (SR) Ca^2+^ uptake (SERCA) and sodium-calcium exchanger (NCX) contribution to Ca^2+^ extrusion were assessed using rapid caffeine application as previously described [19].

#### Assessment of electrophysiological parameters

Cells were studied using a MultiClamp 700A (Axon Instruments) in whole cell patch configuration. Current-voltage relationships for L-type Ca^2+^ current were studied and normalized to cell capacitance, as previously described [19].

#### Assessment of cell volume, t tubules and calcium sparks with confocal microscopy

Cell volume and t-tubule organisation were studied using the membrane binding dye di-8-Anepps (Molecular Probes, Eugene, OR, USA) and the Zeiss Axiovert microscope (Carl Zeiss, Oberkochen, Germany) with an LSM 510 confocal attachment. In addition, local changes in Ca^2+^ levels were assessed using the fluorescent dye Fluo-4 AM (Molecular Probes, USA) and the same confocal microscope as previously described [29].

### Statistical analysis

Statistical comparison of data was performed using one-way analysis of variance followed by Bonferroni post-hoc test for individual significant differences, or Student's t-test where appropriate. All statistical analyses were performed using Prism 4 software (Graph-Pad Software, Inc.) and P<0.05 was considered significant. Data are expressed as mean ±SEM [n], where n is the number of cells unless otherwise specified. All of the experiments were performed using a minimum of four animals, unless otherwise stated.

## Results

### Heart rate

**Figure 1:**
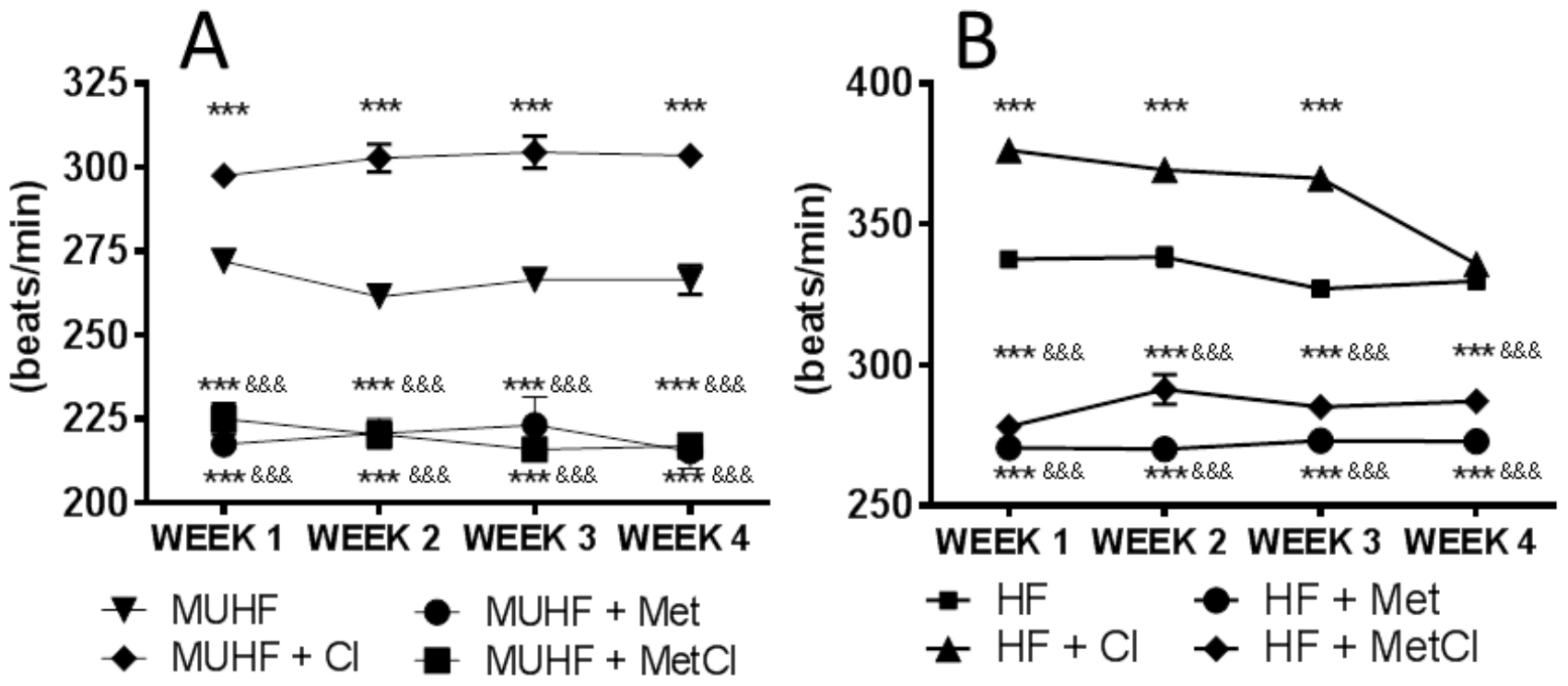
Average HR of mechanically unloaded failing hearts (MUHF) (A) and non-transplanted failing hearts (B) measured using telemetry devices (n = 4 per group). Cl increased HR significantly at all time-points during MU, whereas Met caused equal HR reduction either alone, or in combination with Cl at all time-points during MU. ***P<0.001 vs. MUHF and ^&&&^P<0.001 vs. MUHF+Cl. Cl increased HR in-non-transplanted failing hearts significantly but this effect was lost at week 4. Met caused equal HR reduction in-non-transplanted failing hearts either alone, or in combination with Cl, at all time-points. ***P<0.001 vs. HF and ^&&&^P<0.001 vs. HF+Cl.

There was no difference in average HR between sham-operated and HF group at any time point (mean HR (bpm) at 4 weeks: sham 332±4.8 vs. HF 329±5.5; P>0.05). HR of transplanted failing hearts (MUHF) was significantly lower (∼20%) than that of non-transplanted failing hearts at all time-points (**Figure 1A**). Cl caused a consistent increase in HR (∼12%) at all time-points during MU, with Met either alone, or in combination with Cl reducing HR by approximately 15% (**Figure 1A**). Assessment of HR of non-transplanted failing hearts revealed similar drug effects: Cl caused a significant increase in HR (∼10%) compared to HF group during first 3 weeks of therapy, but this effect was not present at week 4. Met caused significant and equivalent HR reduction (∼20%) either alone, or in combination with Cl, compared to HF group at all-time points (**Figure 1B**).

### LV function

LCA ligation induced LV dysfunction after 12 weeks (time of randomisation), characterised by reduced LVEF (%): sham 83.3±0.8 vs. HF 37.6±0.7; reduced LVFS (%): sham 48.3±0.7 vs. HF 16.6±0.5; and LV chamber dilatation (diastolic diameter (cm)): sham 0.60±0.03 vs. HF 0.98±0.04, P<0.001 for all parameters.

**Figure 2:**
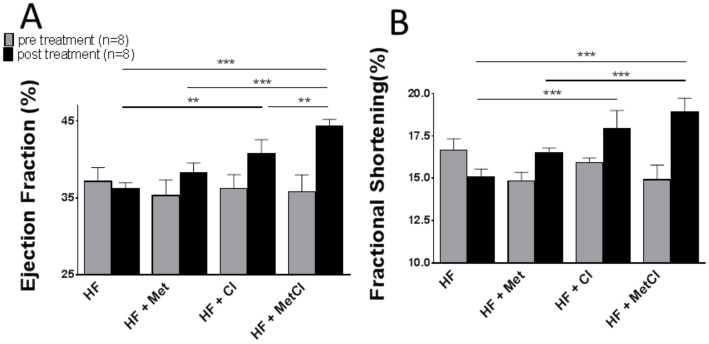
Effect of Cl, Met and combined MetCl therapy on contractile function in non-transplanted failing hearts, EF (A) and FS (B): No difference in baseline values in treatment groups was seen (light grey bars). Average values at end of treatment period are shown (black bars): Cl-treated group showed improved EF and FS compared to untreated HF group, and improvement in EF was further enhanced in MetCl group. Met-induced improvement in EF and FS was not statistically significant. ***P<0.001 (n = 8 per group).

No difference in these parameters was detected between treatment groups prior to commencing therapy (**Figure 2A and B**). *In-vivo* function of transplanted failing hearts was not assessed, but that of non-transplanted hearts was assessed via repeat TTE at the end of the 4 week treatment period. Cl improved contractile function of non-transplanted failing hearts, and improvement in LVEF was enhanced further by combination with Met (**Figure 2A and B**). Met-induced increase in EF and FS was not statistically significant.

### Cardiac and cardiomyocyte size

**Figure 3:**
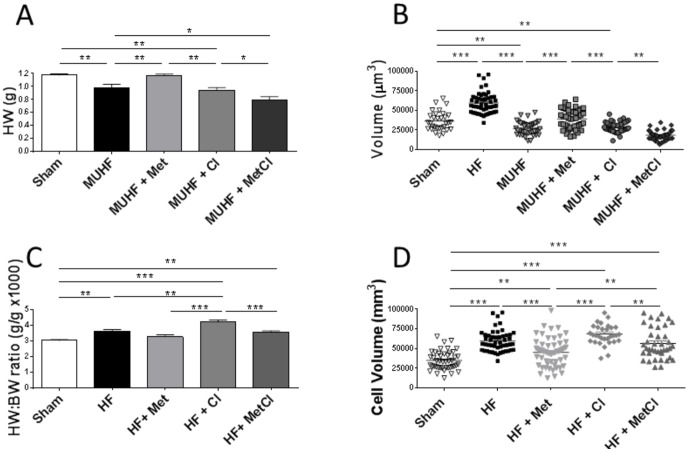
Effect of Cl, Met and combined MetCl treatment on heart weight (HW) (A) and cardiomyocyte volume measured using confocal microscopy (B) during unloading (MUHF). Prevention of MU-induced cardiac and cardiomyocyte atrophy is achieved by Met and not Cl, with combined MetCl therapy increasing atrophy. *P<0.05, **P<0.01 and ***P<0.001, (HW and cardiomyocyte volume data acquired from 8 and 4 hearts per group, respectively). Effect of Cl, Met and combined MetCl therapy on heart weight∶body weight ratio (HW∶BW) (C) and cardiomyocyte volume (D). HF-induced cardiac hypertrophy was enhanced by Cl therapy but this effect disappeared during combined MetCl therapy. HF-induced myocyte hypertrophy was partially attenuated by Met, but this effect was lost during combination MetCl therapy (HW and cardiomyocyte volume data acquired from 8 and 4 hearts per group, respectively).

LCA ligation induced cardiac and cellular hypertrophy, shown by increased total HW, HW∶BW ratio and cardiomyocyte volumes, compared to sham-operated group (**Table 1**). MU of failing hearts induced myocardial atrophy at whole heart and cardiomyocyte level, with both HW and cardiomyocyte volumes falling below those of sham values (**Figure 3A and B**). HW of MUHF group was ∼40% smaller than that of HF group and ∼17% smaller than sham, with cell volume being ∼55% lower than in HF group and ∼26% smaller than sham volumes. Cl had no effect on myocardial atrophy. Met prevented both whole heart and cellular atrophy, restoring mean HW and cardiomyocyte volumes to sham values, whilst combined MetCl therapy worsened MU-induced atrophy (**Figure 3A and B**).

**Table 1:** Anatomical and cell volume data (16 weeks after LCA ligation).

	Sham	HF
*N*	8	8
Body weight (g)	386±5.6	415±6.2**
Heart weight (g)	1.2±0.05	1.6±0.05***
Heart weight/Body weight ×1000	3.0±0.1	3.6±0.1***
Cell volume (µm^3^)	36248±12012	59705±13508***

**P<0.01,

***P<0.001 vs. sham.

In non-transplanted failing hearts Cl augmented cardiac hypertrophy, an effect that disappeared during combined MetCl therapy (**Figure 3C**). At a cellular level Met induced partial regression of cellular hypertrophy, but this effect was also lost during combined MetCl therapy (**Figure 3D**).

### Cardiomyocyte EC coupling

#### MU-induced recovery of deranged EC coupling

**Figure 4:**
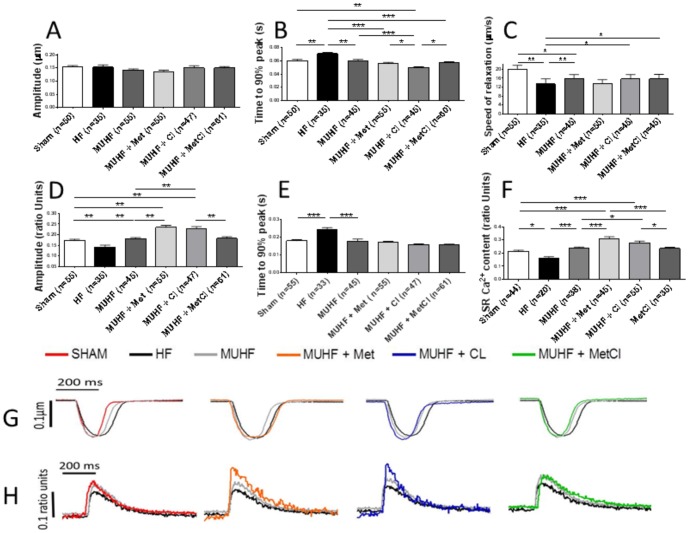
Effects of Cl, Met and MetCl therapy on cardiomyocyte contractility (A–C) and Ca^2+^ handling (D–F) during mechanical unloading (MUHF), measured using Indo-1 and Ionoptix system. Cl's enhancement of MU-induced recovery of speed of sarcomeric contraction (B) and Met's antagonism of MU-induced improvement in speed of relaxation (C) are shown. Cl and Met's enhancement of MU-induced recovery of Ca^2+^ transient amplitude (D), speed of Ca^2+^ release (E) and SR Ca^2+^ content (F), and lack of enhancement during combined MetCl therapy is shown. *P<0.05, **P<0.01 and ***P<0.001. Representative traces of sarcomeric contractions (G) and Ca^2+^ transients (H) are shown.

LCA ligation caused impaired cardiomyocyte contractility and deranged Ca^2+^ cycling: speed of sarcomeric contraction, relaxation and Ca^2+^ release, along with Ca^2+^ transient amplitude were all decreased, sarcomeric contraction amplitude remaining unchanged (**Figure 4 A–E**). MU normalised speed of sarcomeric contraction and this effect was further enhanced by Cl, with Met and MetCl therapy having no added effect (**Figure 4B**). MU partially improved speed of relaxation, and this effect was antagonised by Met, with no additional observed changes following Cl and MetCl treatment (**Figure 4C**). In addition, MU recovered Ca^2+^ transient amplitude, speed of Ca^2+^ release and SR Ca^2+^ content, and this recovery was further enhanced by both Cl and Met mono-therapy equally, but not by combined MetCl treatment (**Figure 4** **D–F**).

**Figure 5:**
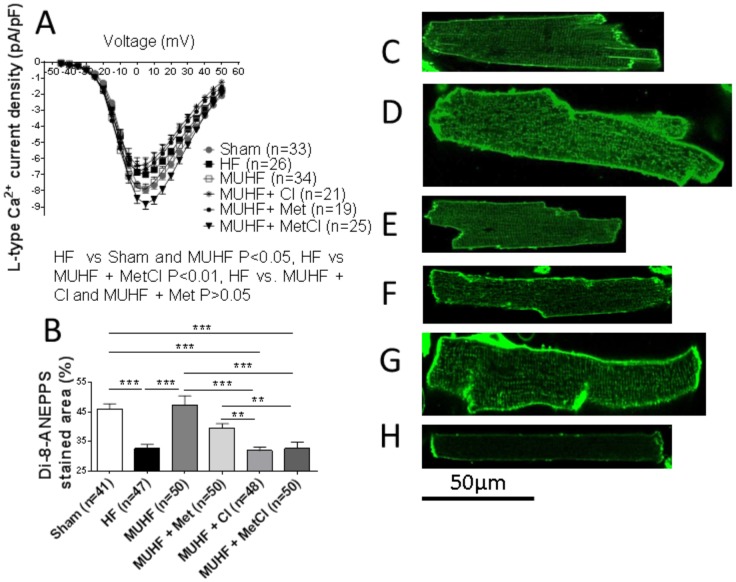
MU-induced recovery of depressed L-type Ca^2+^ current (A) and t-tubule density (B). Maintenance of L-type Ca^2+^ current recovery by combined MetCl therapy, and antagonism of such recovery by Cl or Met mono-therapy (A) is shown, along with antagonism of t-tubule density recovery by all treatments (B). *P<0.05, **P<0.01 and ***P<0.001. Representative di-8-Anepps stained cells from sham (C), HF (D), MUHF (E), MUHF+Cl (F), MUHF+Met (G) and MUHF+MetCl (H) groups are shown. Data for the Met group has been previously published [22] and added here for comparison.

An increased SR Ca^2+^ leak, secondary to altered RyR function, is one mechanism contributing to reduced SR Ca^2+^ load in HF [30]. Ca^2+^ sparks are complex entities regulated by multiple factors, and represent a measure of diastolic SR Ca^2+^ release [29]. Ca^2+^ spark frequency was increased in HF group compared to sham group suggesting increased SR Ca^2+^ leak ((sparks/100 µm/sec): sham 0.80±0.17 [37] vs. HF 3.00±0.34 [40]; P<0.001). MU normalised HF-induced increase in Ca^2+^ spark frequency (sparks/100 µm/sec): MUHF 0.82±0.13 [36] vs. HF 3.00±0.48 [40]; P<0.001. This recovery was unaffected by either Cl or Met mono-therapy, but abolished by combined MetCl therapy (sparks/100 µm/sec): MUHF+Cl 1.67±0.27 [35] vs. MUHF+Met [30] 1.70±0.26; P>0.05, both P>0.05 vs. MUHF, and P<0.001 vs. MUHF+MetCl [30] 3.51±0.36; P<0.001 vs. MUHF and P>0.05 vs. HF. MU also caused recovery of HF-induced depression of L-type Ca^2+^ current and t-tubule density (**Figure 5**). However, this recovery was largely antagonised by all three drug treatments; with only maintenance of L-type Ca^2+^ current recovery occurring in the combined MetCl-treated group (**Figure 5A and B**).

#### Effects of Cl and Met on deranged EC coupling in non-transplanted failing hearts

**Figure 6:**
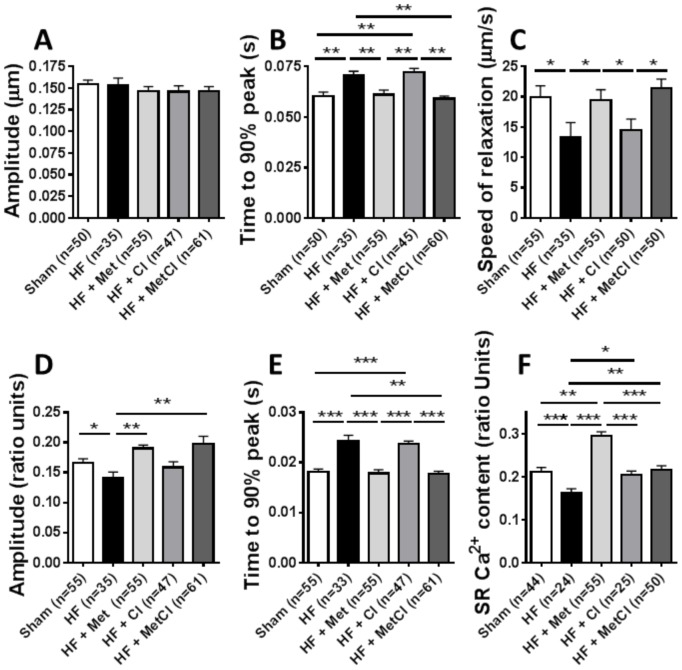
Effects of Cl, Met and MetCl therapy on cardiomyocyte contractility (A–C) and Ca^2+^ handling (D–F) from non-transplanted failing hearts, measured using Indo-1 and Ionoptix system are shown. Full recovery of speed of sarcomeric contraction (B), relaxation (C), Ca^2+^ transient amplitude (D) and speed of Ca^2+^ release (E) caused by Met therapy, either alone or in combination with Cl, and lack of improvement following Cl mono-therapy is shown. Superiority of Met mono-therapy in recovering depressed SR Ca^2+^ content is also shown (F). *P<0.05, **P<0.01 and ***P<0.001.

**Figure 7:**
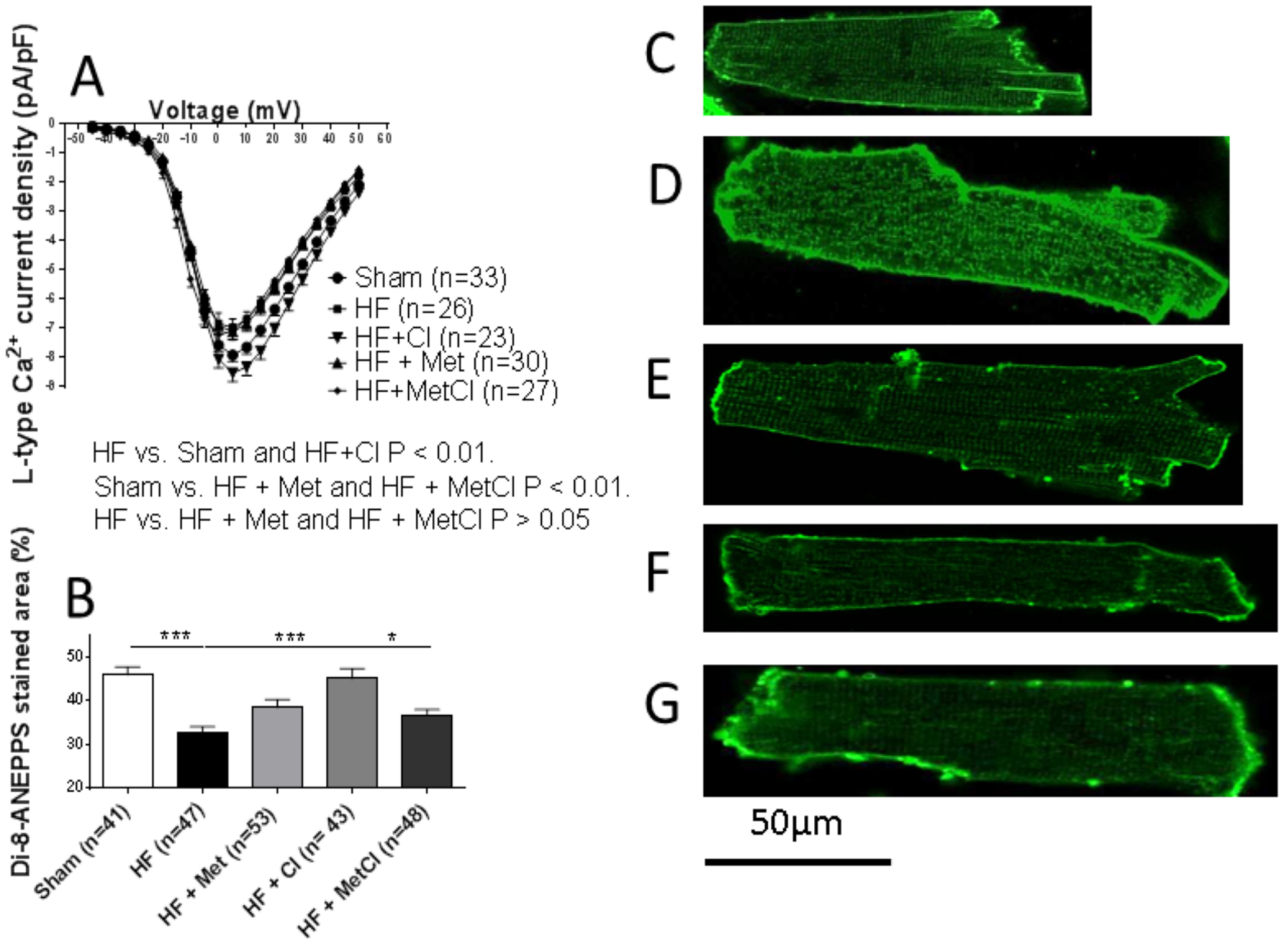
Full recovery of HF-induced depression of L-type Ca^2+^ current (A) and t-tubule density (B) caused by Cl, and lack of improvement in these parameters following Met and MetCl therapy are shown. ***P<0.001. Representative di-8-Anepps stained cells from sham (C), HF (D), HF+Cl (E), HF+Met (F) and HF+MetCl (G) groups. Data for the Met group has been previously published [22] and added here for comparison.

Met therapy, either alone or in combination with Cl normalised speed of sarcomeric contraction, relaxation and Ca^2+^ release, along with Ca^2+^ transient amplitude, while Cl mono-therapy displayed no effect (**Figure 6B–E**). Met, Cl and combined MetCl therapy all recovered SR Ca^2+^ content, with the greatest improvement seen following Met therapy (**Figure 6F**). Cl alone caused significant reduction in spark frequency (HF+Cl 1.10±0.17 [30]; P<0.01 vs. HF), whereas spark frequency was no different to HF in the Met or MetCl-treated groups (HF+Met 2.10±0.22 [30] and HF+MetCl 2.00±0.32 [32], both groups P>0.05 vs. HF). Drug therapies had differing effects on L-type Ca^2+^ current and t-tubule density. HF-induced depression of both these parameters was normalised by Cl; Met therapy alone, or in combination with Cl showed no effect (**Figure 7**).

## Discussion

In the first part of this study we showed that Met but *not* Cl prevented MU-induced myocardial atrophy, with combined MetCl therapy worsening atrophic remodelling. MU induced recovery of deranged EC coupling, with either Cl *or* Met mono-therapy, but *not* combined MetCl therapy, broadly enhancing this recovery. MU-induced recovery of Ca^2+^ transient amplitude, speed of Ca^2+^ release and SR Ca^2+^ content was enhanced by Cl *or* Met mono-therapy equally. However, these positive effects, together with Cl's enhancement of speed of sarcomeric contraction, were lost during combined MetCl therapy, along with MU-induced recovery of Ca^2+^ spark frequency.

The second part of this study showed combined β_2_-AR stimulation (Cl) and β_1_-AR blockade (Met) to improve depressed whole heart function in HF, to a greater degree than either mono-therapy. Cl's improvement in LVEF was enhanced by Met, but the mechanisms underlying this superiority appeared independent of cellular EC coupling. Met and Cl mono-therapy displayed distinct EC coupling effects. Met's effects were more favourable than those of Cl, with lack of evidence supporting additive or synergistic benefit, and more suggestive of selected antagonism during combined therapy. Met alone, and in combination with Cl normalised speed of sarcomeric contraction, relaxation, Ca^2+^ release and Ca^2+^ transient amplitude, and enhanced SR Ca^2+^ content; whereas Cl mono-therapy improved SR Ca^2+^ content to a lesser degree than Met, and normalised deranged Ca^2+^ spark frequency, L-type Ca^2+^ current and t-tubule density, positive effects that were lost during combined MetCl therapy. Of note, undesirable Cl-induced hypertrophic/chronotropic effects were prevented during combined therapy.

These results suggest that A) Cl's success in enhancing functional recovery in LVAD-supported patients is unrelated to myocardial size, and reliant on positive EC coupling effects, B) combined β_2_-AR stimulation +β_1_-AR blockade is likely to be a safe and beneficial HF therapeutic pharmacological strategy and C) either the strategy of β_2_-AR stimulation *or* β_1_-AR blockade is likely to be superior to that of combined β_2_-AR stimulation +β_1_-AR blockade in enhancing functional recovery during LVAD support. This is, however, in contrast with the current clinical experience with β_1_-AR blockade in patients with LVADs and could be explained by the type and degree of adverse remodelling obtained in our models. It cannot be excluded that subpopulations of LVAD patients with specific, lower degrees of dysfunction may benefit from this pharmacological treatment and more clinical studies are warranted.

### Combined β_2_-AR stimulation and β_1_-AR blockade during mechanical unloading

#### Myocardial atrophy

LVAD-mediated unloading of failing ventricles regresses cardiac and cellular hypertrophy [8]. Whether such regression progresses during prolonged clinical LVAD support towards myocardial “atrophy” i.e. decreased cardiac and cell size to sub-normal values, remains controversial [31]. In contrast, convincing experimental data derived mainly from rodent studies employing HATx clearly demonstrate atrophy of normal [13], [28] and failing [11] hearts. It must be emphasised that in this context atrophy relates to decreased size and not function.

A key question surrounding Cl is whether anti-atrophic properties contributed to enhanced recovery rates produced by the Harefield Protocol [18]. Findings of similar myocyte size in both explanted and non-recovered patients treated by this protocol, suggest Cl's pro-recovery effects were unrelated to myocardial size [17]. This present study reinforces this prospect. We show Cl to be ineffective in preventing myocardial atrophy associated with prolonged unloading (4 weeks), and this agrees with previous work [28] in non-failing rat hearts undergoing 2 weeks unloading. In contrast, we previously showed Cl to limit regression of rodent HF myocyte hypertrophy during short-term unloading (1 week) [19]. However, such a brief period of MU, during which atrophic remodelling is regarded as being sub-maximal [11], [13] was considered inadequate, and a poor representation of prolonged clinical LVAD support.

As we have previously shown [22], Met prevented myocardial atrophy, but this effect was lost during combined MetCl therapy with actual worsening of atrophy. The latter observation is unexpected, if compared with the effects of the combined therapy in normally-loaded hearts; this can be due to additional detrimental consequences of mechanical unloading and require to be further studied. Atrophic remodelling is complex and the multiple pathways involved poorly defined [32]. The ubiquitin proteosome, calpain, lysosomal proteolysis and authophagy systems, and mTOR IGF-1/PI3K/AKT and ERK-1 pathways are all altered during MU [32]. These growth regulatory pathways, along with the TGF-β, CAMKII and calcineurin/NFAT hypertrophic signalling pathways, known components under β-AR influence [33], may represent Met's route of action, and augmentation of myocyte number (not assessed in this study) via regenerative mechanisms is another possibility. This finding is particularly important as, to date, no pharmacotherapy has proven effective in attenuating myocardial atrophy, with success via haemodynamic loading strategies alone [34], re-emphasising the critical importance of load in regulation of cardiac mass [13]. Such loading, potentially brought about by HR reduction and subsequent augmentation of LV filling, may have driven Met's anti-atrophic actions; but the lack of effect during combined MetCl therapy, despite an equivalent reduction in HR, makes this mechanism unlikely.

#### EC coupling during mechanical unloading

LVAD support induces reversal/alteration in expression and function of numerous Ca^2+^ handling elements adversely remodelled during HF. Recovery of SR Ca^2+^ content, L-type Ca^2+^ current fast inactivation and Ca^2+^ transient amplitude is seen [35], and these parameters show positive correlation with functional recovery in patients treated with the Harefield Protocol [17]. LVAD-induced enhanced SERCA 2A expression [36] and stabilisation of RyR function [37] are potentially involved mechanisms.

In the present study, MU recovered HF-induced deranged EC coupling with normalisation of Ca^2+^ transient amplitude, speed of Ca^2+^ release and sarcomeric contraction, SR Ca^2+^ content, Ca^2+^ spark frequency, L-type Ca^2+^ current amplitude and t-tubule density. Drug effects on this recovery were not clear-cut but overall, the positive effects of either mono-therapy disappeared during combined therapy. MU-induced recovery of Ca^2+^ transient amplitude, speed of Ca^2+^ release and SR Ca^2+^ content, proven markers of recovery during LVAD support [17], were equally enhanced by Cl and Met mono-therapy, with Cl also enhancing speed of sarcomeric contraction. These effects were lost during combined therapy along with MU-induced recovery of RyR function (normalised Ca^2+^ spark frequency).

Cl's effects may originate from improved myofilament sensitivity [19] and SERCA2a expression [28] previously shown during MU. MU is associated with increased β_2_-AR mRNA expression [21] and adenoviral mediated β_2_-AR overexpression with improved function following MU [38]. These findings, coupled with results from our study, re-enforce the rationale for the use of Cl/β_2_-AR agonists during LVAD support. Specific study of β_1_-AR blockade during MU is lacking. Hence, mechanisms driving Met's effects are speculative. Enhancement of MU-induced recovery of β-AR responsiveness [35] and/or RyR and SERCA/phospholamban function/phosphorylation/expression [36], [37], proven actions of β-blockers in non-unloaded failing myocardium [4] may be involved. Despite mainly beneficial effects during MU, the antagonism of L-type Ca^2+^ current and t-tubule density recovery highlight potentially unwanted effects of Cl and Met, the significance of which requires further investigation.

### Combined β_1_-AR blockade and β_2_-AR stimulation in heart failure

#### β-adrenergic signalling

Deranged β-adrenergic signalling is instrumental in HF pathogenesis [4]. Selective down-regulation/de-sensitisation of β_1_-ARs promotes relative augmentation of β_2_: β_1_-ARs ratio and signalling [4], [39]. Down-regulation of β_1_-AR signalling is thought protective against heightened sympathetic stimulation, and the clinical efficacy of β_1_-AR blockade is proven [4], [6], [23].

Augmented β_2_-AR signalling is also considered cardio-protective [24], and the idea that β_2_-AR agonism may be beneficial in clinical HF is long-standing [40], but remains controversial and poorly studied in humans. Undesirable β_2_-AR agonist effects such as elevation of HR and arrhythmia risk shown by certain studies [41], [42], along with fears over increasing LV mass, both independent prognostic risk factors in cardiac disease [43], [44] tempered enthusiasm for using β_2_-AR agonists in HF therapy. These factors, coupled with expanding evidence supporting β-blocker efficacy [4], [6], [23], and the phenomenon of β_2_-AR agonist tachyphylaxis, rendered the notion of therapeutic β_2_-AR agonism illogical and unsound. Hence, little clinical data exists regarding β_2_-AR agonist administration within this context, with data derived from a few small, non-randomised studies in HF patients [45]–[46] and chronic lung disease patients with HF, receiving β_2_-AR agonist therapy [42], [47]. Results are unclear, showing both sustained [42], [45] and transient [46] improvements in cardiac function, as well as negative functional effects [47].

This study re-enforces the concept that β_2_-AR agonism represents a beneficial HF therapeutic strategy, albeit alongside that of clinically proven β_1_-AR blockade [24]. Cl's undesirable effects of cardiac hypertrophy and tachycardia were inhibited by Met with no evidence of pro-arrhythmic behaviour (**Figure S1 in Methods S1**). Combined MetCl therapy displayed superior improvement in whole heart systolic function to either mono-therapy, although a cellular mechanism explaining this superiority was not apparent. Met and Cl showed different positive EC coupling effects with evidence of selected antagonism rather than synergy. Met's improvement of cellular contraction, relaxation, Ca^2+^ release, Ca^2+^ transient amplitude, and SR Ca^2+^ was not enhanced by Cl. T-tubule disruption uncouples the normally tight relationship between L-type Ca^2+^ channels and RyRs, reducing EC coupling efficiency [48]. Cl improved this coupling shown by recovery of L-type Ca^2+^ current and t-tubule density, along with RyR function (normalised Ca^2+^ spark frequency). Such improvements appeared not to alter cellular contractility, but were abolished by Met.

Additive anti-apoptotic effects of either Cl [20] or fenoterol [24] in combination with Met, demonstrated by previous rodent HF studies [20], [24] may be responsible for the whole heart functional effects. Such a mechanism is plausible, as opposing actions of the two β-adrenergic signalling pathways on cell survival is recognised; β_1_-AR signalling broadly promoting apoptosis [25] and β_2_-AR signalling driving improved myocyte viability [26], [27]. Restoration of Ca^2+^ handling protein expression e.g SERCA2a or RyR [18], [20], myo-filament sensitivity [19] and myocardial stiffness/diastolic function [49] represent other properties of Cl and Met, that may underpin the augmented function seen during combined therapy; and afterload (blood pressure (BP)) reduction is another. However, at similar doses to those used in this study, chronic Met [20], [50], Cl [20] or combined therapy [20] has not been seen to significantly alter BP. β_2_-AR agonist tachyphylaxis is recognised following chronic administration [24], [45]. Temporal functional effects were not assessed in this study, and whether tachyphylaxis occurred is unknown. The loss of Cl's chronotropic effect at week 4 suggests it may have done. In a longer rodent HF study (1 year), Met was shown to prevent β_2_-AR agonist (fenoterol) tachyphylaxis (caused by decreased β_2_-AR density), producing extension of fenoterol's positive functional effects. Such a process may also explain the findings from our, albeit shorter study.

## Conclusions

In conclusion, our study shows that Met but not Cl prevented MU-induced myocardial atrophy, with atrophic worsening during combined therapy. Cl and Met broadly enhanced MU-induced recovery of deranged EC coupling, but this enhancement was lost during combined therapy. These results suggest that A) Cl's success in enhancing functional recovery in LVAD-supported patients is unrelated to myocardial size and reliant on positive EC coupling effects and B) either the strategy of β_2_-AR stimulation *or* β_1_-AR blockade is likely to be superior to that of combined β_2_-AR stimulation +β_1_-AR blockade in enhancing functional recovery during LVAD support. Such findings warrant further investigation.

In the second part of this study, we show for the first time in a model of established rodent HF that combined β_2_-AR simulation (Cl) +β_1_-AR blockade (Met) displays superior functional effects to either mono-therapy, and undesirable Cl-induced hypertrophic/chronotropic effects are prevented by Met. Mechanisms underlying this superiority appeared independent of cellular EC coupling and require further investigation. These results suggest that this pharmacological strategy is likely to be safe and beneficial in HF therapy.

## Limitations

Met was used in this study to facilitate comparison with previous studies using this rodent HF model. Bisoprolol and not Met is used in the Harefield Protocol. As such, findings from this study may not be directly applicable to this specific clinical scenario.

## Supporting Information

Methods S1 **A detailed description of Material and Methods is provided.** The file also contains Figure S1 showing the ventricular ectopic (VE) rate in sham, failing and treated failing hearts.
(DOC)
