# Supplementary material for: Impact of Combined Clenbuterol and Metoprolol Therapy on Reverse Remodelling during Mechanical Unloading
Source: PLoS One. 2014 Sep 30;9(9):e92909. doi: 10.1371/journal.pone.0092909 (PMC4181979; doi:10.1371/journal.pone.0092909)
Supplement: Methods S1 — A detailed description of Material and Methods is provided. The file also contains Figure S1 showing the ventricular ectopic (VE) rate in sham, failing and treated failing hearts. (DOC) [file pone.0092909.s001.doc]

**Materials and Methods S1**

***In-vivo* assessment of heart rate of failing and mechanically unloaded failing hearts**

4 animals per treatment group received implantable ECG telemetry transmitters (CA-F40 Data Sciences International, Minneapolis, MN) on day 0 of the 4 week treatment period, for *in vivo* HR and arrhythmia studies. Animals were anaesthetised with isoflurane (5% induction, 1.5% maintenance) mixed with O_2_ (2-3 L/min) and telemetry devices were implanted subcutaneously (SC). Electrodes were placed (A) in the lead 2 position SC over the chest wall in non-transplanted hearts or (B) intra-abdominally at time of surgery, secured to right atrium and LV apex of the transplanted hearts. Animals underwent HR assessment with continuous 24 hour ECG recording at weekly intervals after implantation during the 4 week treatment period (Day 7, 14, 21 and 28). *In vivo* ECG recordings were acquired using Dataquest ART 3.1 software (Data Sciences International, Minneapolis, MN), and offline HR/arrhythmia analysis performed using ECG-Auto 2.4 software (EMKA, France). PR, QRS, QT intervals, mean HR and mean ventricular ectopic (VE) rate were automatically evaluated. A VE was defined as an abnormal irregular premature supraventricular QRS, without a preceding P wave, with prematurity exceeding the normal variation of the RR interval by 60% [1].

**Heart harvest and macroscopic analysis**

4 weeks after commencement of therapy (drug alone or in combination with MU) animals were weighed, and then sacrificed via schedule 1 cervical dislocation. Hearts were rapidly excised from thorax or abdomen and cardiac mass was measured after the heart was blotted on paper. Heart weight to body weight (HW:BW) ratio was calculated for hearts that had not been mechanically unloaded, and absolute values of cardiac mass were utilised for all hearts which had undergone MU. In each group of 8 animals, 4 hearts were utilised for acute cellular experiments and 4 hearts were processed for histological analysis.

**Cardiomyocyte studies**

LV cardiomyocytes were isolated by standard enzymatic digestion for 8-10 minutes using collagenase type 2 (1mg/ml, Worthington, USA) and hyaluronidase (0.6 mg/ml, Sigma, England), as previously described [2]. Cardiomyocytes were used within 6h and those used for study were randomly selected and only excluded if they did not possess rod shaped morphology or were contracting spontaneously.

***Assessment of sarcomere shortening and Ca^2+^handling***

Cardiomyocytes were placed on a superfusing chamber (~70 µl) positioned on the stage of an inverted microscope (TE 200). Cell adhesion was improved by application of a thin coating of laminin (Sigma, England) to the surface of the chamber. Cytoplasmic Ca^2+^ was measured using Indo-1 AM (Molecular Probes, USA), a Ca^2+^ sensitive, single-excitation dual emission fluorescent dye. Cells were superfused with a Normal Tyrode (NT) solution containing (in mM): 140 NaCl, 6 KCl, 1 MgCl_2_, 10 glucose, 10 HEPES, 1 CaCl_2_; pH 7.4; field stimulated at 1 Hz and illuminated by red light (wavelength of 600 nm), allowing the cardiomyocyte in view to be studied for simultaneous measurement of Indo-1 fluorescence and sarcomere shortening using an Ionoptix system (Ionoptix Corporation, USA), as previously described [3]. Cardiomyocytes were loaded with 10 µM of the AM form of the indicator for 30 min at room temperature. The supernatant was subsequently discarded and replaced by fresh buffer solution. Cells were stored in the dark at room temperature and used within 6 h. The assessment of sarcoplasmic reticulum (SR) Ca^2+^ content was achieved by stimulation of cells to steady-state contraction upon which stimulation was stopped, followed by the rapid application of 20mM caffeine in Na^+^/Ca^2+^ free solution (in mM): 140 LiCl, 6 KOH, 1 MgCl_2_, 10 glucose, 10 HEPES, 0.1 EGTA; pH to 7.4 with 1M LiOH, for 5 seconds, as previously described [3]. Peak amplitude of caffeine-induced Indo-1 transient was taken as an index of SR Ca^2+^ content. Contribution of the sodium-calcium exchanger (NCX) to Ca^2+^ extrusion was assessed using the time constant (τ) , derived by fitting a mono-exponential curve on the Ca^2+^  transient decay phase.

***Assessment of electrophysiological parameters***

Cells were studied using a MultiClamp 700A (Axon Instruments, USA) in whole cell patch configuration, as previously described [4]. The pipette resistance was ~2–3 MΩ, and the pipette-filling solution contained the following (in mM): 115 caesium-aspartate, 20 tetraethylammonium-chloride, 10 EGTA, 10 HEPES, and 5 Mg ATP, pH 7.2. The external solution contained the following (in mM): 140 NaCl, 10 glucose, 10 HEPES, 1 CaCl_2_, 1 MgCl_2_, 6 CsCl, pH 7.4. Current-voltage relationships for L-type Ca^2+^ current were built using 450-ms depolarization steps from a holding potential of -40 mV (range: -40 mV to +40 mV, in 5-mV increments) at 1 Hz. After that Cd^2+^ (200 µM) was applied, and the protocol repeated. Subtracted currents were obtained and normalized to cell capacitance. All experiments were conducted at 37°C.

***Assessment of cell volume, t-tubules and Ca^2+^ sparks with confocal microscopy***

The membrane binding dye di-8-Anepps (Molecular Probes, USA) was utilised to assess cell volume and t-tubule structure, as previously described [5]. Isolated cells were loaded with di-8-Anepps (10 µM) for 10 minutes, and then washed in buffer solution. The experimental chamber was mounted on the stage of a Zeiss Axiovert microscope (Carl Zeiss, Germany) with an LSM 510 confocal attachment, and myocytes were observed through a Zeiss EC Plan-NeoFluar X40 oil immersion lens (numerical aperture 1.3). Di-8-Anepps was excited using the 488-nm line of an argon laser, and the emitted fluorescence was collected through a 505-nm long-pass filter. A focal plane that excluded the nuclei was selected for high-resolution imaging of the t-tubule structure. Lower resolution Z-stack images of the same cells were used to assess the density of the t-tubule network and cell volume. Z-stack images were analysed using a custom-written macro in ImageJ (U.S. National Institutes of Health; http:// rsb.info.nih.gov/ij/).

Local changes in Ca^2+^  levels were assessed using the fluorescent dye Fluo-4 AM (Molecular Probes, USA), as previously described [5], and cells were analysed using the same confocal microscope described above. Cells were incubated with Fluo-4 AM (10µm) for 20 min, then washed and allowed to de-esterify for at least 30 min before use. Cells were superfused with Normal Tyrode and field stimulated. After a period of 30s of quiescence, line scans were collected. Analysis was performed using custom-written routines in MATLAB R2006b (The MathWorks, Inc.) following the threshold-based algorithm for automatic Ca^2+^ spark detection of Cheng *et al* [6]*.* The detection criteria for Ca^2+^ sparks was set at 3.8 standard deviations above the background noise and Ca^2+^ spark frequency was obtained from the line scans. Mean Ca^2+^ spark frequency was compared between groups.

**Statistical analysis**

Statistical comparison of data was performed using one-way analysis of variance followed by Bonferroni post-hoc test for individual significant differences or Student’s t-test where appropriate. All statistical analyses were performed using Prism 4 software (Graph-Pad Software, USA) and P<0.05 was considered significant. Data are expressed as mean ± SEM [n], where n is the number of cells, unless otherwise specified. All of the experiments were performed using a minimum of four animals, unless otherwise stated.

**Reference List S1**

1. Walker MJ, Curtis MJ, Hearse DJ, Campbell RW, Janse MJ, et al. (1988) The Lambeth Conventions: guidelines for the study of arrhythmias in ischaemia infarction, and reperfusion. Cardiovasc Res 22:447-55. doi: 10.1093/cvr/22.7.447
2. Hering S, Bodewei R, Wollenberger A (1983) Sodium current in freshly isolated and in cultured single rat myocardial cells: frequency and voltage-dependent block by mexiletine. J Mol Cell Cardiol 15:431-44. [doi.org/10.1016/0022-2828(83)90263-8](http://dx.doi.org/10.1016/0022-2828(83)90263-8)
3. Soppa GK, Lee J, Stagg MA, Felkin LE, Barton PJ, et al. (2008) Role and possible mechanisms of clenbuterol in enhancing reverse remodelling during mechanical unloading in murine heart failure. Cardiovasc Res 77(4):695-706. doi: 10.1093/cvr/cvm106.

4. Terracciano CM, Philipson KD, MacLeod KT (2001) Overexpression of the Na(+)/Ca(2+) exchanger and inhibition of the sarcoplasmic reticulum Ca(2+)-ATPase in ventricular myocytes from transgenic mice. Cardiovasc Res 49:38-47. doi: 10.1016/S0008-6363(00)00205-4

5. Ibrahim M, Al Masri A, Navaratnarajah M, Siedlecka U, Soppa GK, et al. (2010) Prolonged mechanical unloading affects cardiomyocyte excitation-contraction coupling, transverse-tubule structure, and the cell surface. FASEB J 24:3321-3329. doi: 10.1096/fj.10-156638

1. Cheng H, Song LS, Shirokova N, Gonzalez A, Lakatta EG, et al. (1999) Amplitude distribution of calcium sparks in confocal images: theory and studies with an automatic detection method. Biophys J 76:606-17. doi: 88/4/1491 [pii];10.1152/physrev.00030.2007

**Figure S1**. The ventricular ectopic (VE) rate in sham, failing and treated failing hearts is shown. Incidence of VEs was unaffected by Cl treatment, but was returned to almost sham levels by Met treatment, both alone and in combination with Cl treatment at all time-points. Week 2 time-point shown. ***P<0.001.
